# Monitoring and addressing the long-term impacts of the COVID-19 pandemic on women in academic science, engineering, and medicine

**DOI:** 10.1073/pnas.2304603120

**Published:** 2023-05-01

**Authors:** Marie Harton, Terri Goss Kinzy, Ashley Bear

**Affiliations:** ^a^Program Officer, National Academies of Sciences, Committee on Women in Science, Engineering, and Medicine, Washington, DC 20001; ^b^President, Illinois State University, Normal, IL 61790; ^c^Director, Committee on Women in Science, Engineering, and Medicine, National Academies of Sciences, Engineering, and Medicine, Washington, DC 20001

## Hard-Won Progress Could Be Lost

Between 1970 and 2019, the representation of US women in Science, Technology, Engineering, and Mathematics (STEM) went from 8 to 27% (1), but these hard-won strides could be lost if higher education and research leaders do not monitor and address the long-term impacts of the COVID-19 pandemic, particularly for those vulnerable populations hit hardest by the pandemic, such as women at the early stages of their scientific careers, and for women of multiple marginalized identities, such as women of color. Today, stories of the COVID-19 pandemic causing careers to be jeopardized, delayed, or lost are commonplace. In 2021, the National Academies released a report that examined the impacts of the COVID-19 pandemic on women’s research careers within the first year of the pandemic, and the results were sobering. The report found that the COVID-19 pandemic had overall negative effects on women in academic science, technology, engineering, mathematics, and medicine in myriad ways, including reduced productivity, new strains as a result of blurred boundaries between home and work, lost opportunities for networking and community building, high burnout rates, and poor mental well-being. Furthermore, the report documented a profound effect on those with family caregiving responsibilities. The report stressed that the disruptions caused by the pandemic inhibited the engagement, experience, and retention of women in academic science, engineering, and medicine. To quote one faculty member: “The pandemic has radically changed everything… Even if there were enough hours in the day, I simply do not have the mental bandwidth to be a full-time homeschooling mom, housekeeper, instructor, researcher, and family member.” (2)

But even as the pandemic has been devastating in so many ways, it may also offer opportunities to reimagine and rebuild the research enterprise in more equitable, fair, and inclusive ways. To quote Marcia McNutt, President of the National Academy of Sciences, “for some of the longstanding issues, the pandemic has served to create a sense of greater urgency to take action.” Indeed, many of the challenges facing women in STEM in the context of the pandemic are not new, but rather have simply been exacerbated and made more visible. Some preliminary research also suggests that some changes to modes of work that have emerged as a result of the pandemic may have some benefits for promoting greater diversity, equity, and inclusion in STEM. For instance, the normalization of virtual and hybrid conferences, meetings, and classes has the potential to create more inclusive opportunities for participation for a broader range of individuals and groups. That said, even as we consider these possible positive outcomes, it remains important that leaders in higher education, government, industry, and nonprofit institutions approach changes to policy and practice carefully and with plans to evaluate their impact over the long term.

## A Workshop to Explore the Research Needed

As academic institutions and government agencies look toward the future, many are grappling with how and what to monitor in order to mitigate the negative long-term impacts of the COVID-19 pandemic, particularly on individuals from groups underrepresented in the sciences. To aid institutions in these forward-looking efforts, the National Academies of Science, Engineering, and Medicine (NASEM) convened a virtual workshop on March 23 to 24, 2022, to explore the long-term impact of COVID-19 on the future careers of women in STEM. The workshop was planned by an expert committee that included Suzanne Barbour, The University of North Carolina at Chapel Hill; Leslie Gonzales, Michigan State University; Elena Fuentes-Afflick, University of California, San Francisco; Jerry Jacobs, University of Pennsylvania; Adia Harvey Wingfield, Washington University in St. Louis; and the committee chair, Terri Goss Kinzy, Illinois State University. Marie Harton, National Academies, served as the study director.

**Fig. 1.**
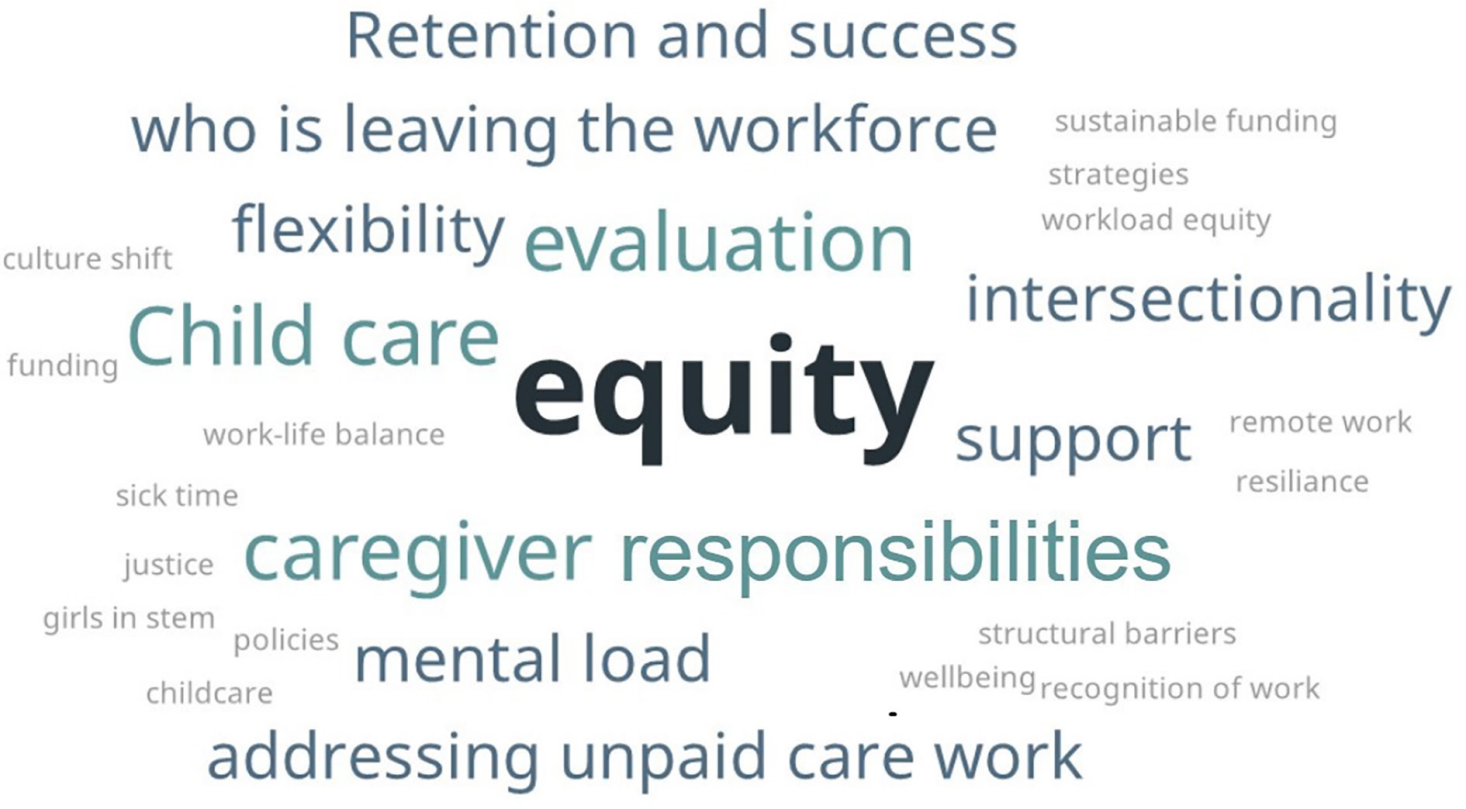
Key topics that a national research agenda on long-term COVID-19 impacts should explore.

The 2-d workshop convened experts and leaders to inform a research agenda that could help institutions monitor and mitigate the long-term impacts of the COVID-19 pandemic on women in STEM fields, while also considering how approaches to working, collaborating, and networking that developed in reaction to the pandemic may serve either to support or hinder greater equity, diversity, and inclusion in STEM. Workshop audience participants represented multiple sectors (i.e., higher education, government, and nonprofit) and various career stages (e.g., professors, graduate students, program officers, and policy advisors). An informal audience poll collected insights on the key topics that a national research agenda on long-term COVID-19 impacts should explore (Fig. 1). For a detailed account of the workshop discussions, please see the published workshop proceedings in brief (3).

Expanding on the 2021 NASEM consensus study report, the workshop was organized into panel sessions focused on a number of important topics related to long-term COVID-19 impacts. Some speakers primarily discussed future-looking research questions, while others also suggested key actions for institutions to implement and evaluate current practices in academic STEM environments. Several key themes and action items emerged throughout the two-day workshop across the panel discussions.

## The Critical Need for Disaggregated Data Collection

One major theme across the workshop presentations was the importance of taking an intersectional approach to data collection efforts and institutional decision-making that considers how some people, as a result of their intersecting identities, experience multiple, unique forms of discrimination or systemic disadvantage.^*^ Christa Porter, Kent State University, urged institutions to disaggregate data by race, gender, and career status to avoid conflation between identities, particularly for women of color; and Lori Patton Davis, The Ohio State University, noted that intersectional identities will highlight larger problems with promotion and tenure processes across the board. Related to tenure and promotion processes, Ethel Mickey, University of Massachusetts Amherst, encouraged institutions to recalibrate faculty evaluation systems by matching their expectations to the relative resources and opportunities available to faculty. Joya Misra, University of Massachusetts Amherst, discussed the need to retrain evaluators to avoid assessing an individual’s accomplishments based solely on their own personal experience with the pandemic. Misra added, “What we are asking is, think about people’s productivity in the context of their workload and the context in which they carried out the work.” This can also include assessing the impact of tenure and promotion clock extensions implemented at almost all universities and disaggregating this data by race and gender. Margaret Sallee, University of Buffalo, added to the point of rethinking evaluation structures. “We are so concerned about particular metrics, but it is time to rethink the metrics,” she said. “[The current structure] penalizes anyone who is not the ‘Ideal Worker.’” Speakers also pointed out the need for more qualitative research, in addition to quantitative research, to better understand individual experiences and delve deeper into the supports and resources faculty need to be productive within their specific institutional environment.

## A Systemic Response to Support Family Caregivers in STEM

Another prominent theme of the workshop was family caregiving and the need for systemic responses to COVID-19 to support this group. Sarah Demaske, Pennsylvania State University, noted, “Many of the responses to COVID-19 in the academy and beyond have focused on individual-level accommodations. Our research suggests the need for systemic responses. Individually tailored responses will only serve to increase bias.” Mary Blair-Loy, University of California San Diego, discussed the difficulty in counteracting cultural schemas in STEM that foster beliefs that “mothers are more distracted and less devoted to science, even though on average, mothers’ productivity is equal to others.” She suggested that institutions broaden paid leave policies to caregivers of children, the elderly, or infirmed to lessen the stigma around these policies. Larissa Mercado-López, California State University, Fresno, stressed the importance of leadership accountability and institutional-wide responses. “I always go back to the need to institutionalize these commitments by showing how caregiver retention moves the needle on institutional goals,” she said. “We should mandate that leaders report on their success, with public-facing data, which would help in accountability.”

## Mental Health and Well-Being

In an informal poll, the audience indicated that “burnout” was one of the most concerning long-term impacts of the pandemic. Sallee remarked that so many faculty and graduate student parents were overwhelmed, and she articulated concern over the long-term career advancement for caregiving women who submitted fewer proposals and articles during the onset of the pandemic. Broadening this discussion, Craig Ogilvie, Montana State University, highlighted research from his group that suggested that 30% of graduate students are experiencing posttraumatic stress symptoms due to pressures by faculty advisors to embody the “Ideal Worker” framework, which is characterized by constant professional availability and visibility and unwavering work devotion. Ogilvie noted that institutional leaders should ask how their institution can make graduate education more responsive and develop educational practices that consider the student holistically. Citing remote work as an example, speakers also noted that the pandemic has heightened awareness of the range of individuals’ needs and of the supportive practices suited to meet them.

## Don’t Strive to “Return to Normal”

Since the pre-COVID-19 world was riven with inequality, the attendees concluded that institutions should not aspire to a “return to normal.” Mary Frank Fox, Georgia Institute of Technology, urged “Consider the work settings, and then consider how the settings may have faded during the pandemic. Take that as an opportunity to reshape settings in significant and inclusive ways.” Relatedly, Heather Shipley, University of Texas San Antonio, encourages universities to take the opportunity to reassess their practices during the pandemic, especially as it relates to flexible work. “They can come out on the other side stronger and better organizations than they were,” she stated. To truly impact lasting equity and a full recovery, it is clear that systematic, comprehensive, and broadly disseminated research and not individual studies or action at institutions is required. Funding agencies, publishers, and academic societies have a significant amount of data to contribute to the assessment of COVID-19 impacts on a broad scale.

The lessons learned from this workshop will inform future work of the National Academies. For example, the National Academies’ Committee on Women in Science, Engineering, and Medicine has launched a consensus study report on policies and practices in support of family caregivers in science, technology, engineering, mathematics, and medical (STEMM) careers, which includes in its scope students and professionals caring for children as well as for adults. The report will be available in the spring of 2024 and will offer recommendations for government and research institutions on how to take actionable steps to improve retention and advancement of family caregivers in STEMM and advance inclusive excellence in the wake of the pandemic. This effort will build from the key insights that came to light at the 2022 workshop. The consensus study, like the workshop, seeks to address a suite of longstanding issues that have undermined gender equity in the sciences and that have been exacerbated by the pandemic. The goal, as many speakers at the workshop pointed out, is not to return to the conditions before the pandemic, but to rebuild our institutional structures with equity at the foundation.

## Footnotes

^*^The concept of intersectionality was originally developed by legal scholar and civil rights leader Kimberlee Crenshaw. Crenshaw demonstrated that Black women were excluded from legal narratives about racial justice, which largely focused on Black men’s experiences, and gender equality, which largely focused on White women’s experiences. For more information, see National Academies of Sciences, Engineering, and Medicine, “Transforming Trajectories for Women of Color in Tech” (The National Academies Press, Washington, DC, 2022; https://doi.org/10.17226/26345).

## References

[1] U. S. Census Bureau (2021). Women Making Gains in STEM Occupations but Still Underrepresented. https://www.census.gov/library/stories/2021/01/women-making-gains-in-stem-occupations-but-still-underrepresented.html.

[2] National Academies of Sciences, Engineering, and Medicine, The Impact of COVID-19 on the Careers of Women in Academic Sciences, Engineering, and Medicine (The National Academies Press, Washington, DC, 2021), 10.17226/26061. 33705087

[3] National Academies of Sciences, Engineering, and Medicine, Long-Term Impacts of COVID-19 on the Future Academic Careers of Women in STEM: Proceedings of a Workshop—in Brief (The National Academies Press, Washington, DC, 2022), 10.17226/26687.

